# DESIGNING FOR LATER LIFE: SOCIO-GERONTOLOGICAL INSIGHTS IN CONVERSATIONS WITH AGE-TECH EXPERTS

**DOI:** 10.1093/geroni/igae098.0832

**Published:** 2024-12-31

**Authors:** Stephen Katz

**Affiliations:** Trent University, Toronto, Ontario, Canada

## Abstract

This presentation summarizes original research data based on interview conversations with 11 international experts, consisting of designers, innovators and leaders in the gerontechnological field. Their experiences reveal important insights about the creation, production and distribution of care technologies for older adults, and how priorities are imagined for the future. Four main themes emerged from the research that point to a need for wider gerontological and sociological engagement. First, while the experts are aware of the increasing demand for technologies delivering monitoring, robotic, assistive, connective and mobility services, their socio-gerontological research about older adults on issues such as personal privacy is often limited. Second, the experts are challenged to recognize where ageist stereotypes of frailty and decline become scripted into their products. However, a clear protocol for avoiding negative imagery is not always available across age-tech enterprises, even as such imagery affects the appeal and promotion of their products. Third, the benefits of co-design and collaborate participation between designers and older users are not well understood, thus opportunities are lost to create age-friendly, affordable and livable products from the perspective of the users. Fourth, the conditions under which technologies become successful require the labor of care-givers, family members, residential staff and technical repair and maintenance technicians. Thus, understanding their roles should be an essential part of the design process. Conclusions advocate greater ties and co-learning amongst gerontologists, sociologists and age-tech experts, and attention to critical and grounded research on the material realities and challenges of technological adaptation for older adults.

